# A Study on Risk Factors for Bovine Tuberculosis in the Disease-Free Regions of Italy

**DOI:** 10.3390/pathogens15060636

**Published:** 2026-06-15

**Authors:** Giorgio Galletti, Sara Salvato, Stefania Calò, Maria Ottaiano, Maria Beatrice Boniotti, Marco Tamba

**Affiliations:** 1 Istituto Zooprofilattico Sperimentale Della Lombardia e dell’Emilia Romagna “Bruno Ubertini”, 25124 Brescia, Italy; giorgio.galletti@izsler.it (G.G.); sara.salvato@izsler.it (S.S.); stefania.calo@izsler.it (S.C.); mariabeatrice.boniotti@izsler.it (M.B.B.); 2 Istituto Zooprofilattico Sperimentale del Mezzogiorno, 80055 Portici, Italy; maria.ottaiano@izsmportici.it

**Keywords:** Bovine Tuberculosis, *Mycobacterium tuberculosis* complex, risk factor, surveillance

## Abstract

Animal tuberculosis in cattle (TB) has been controlled in many European countries through long-standing eradication programs, yet sporadic breakdowns continue to occur in officially tuberculosis-free (OTF) areas, challenging the sustainability of disease freedom. This study aimed to identify and quantify herd-level and area-level risk factors associated with TB occurrence in Italian OTF regions in order to support risk-based surveillance strategies. A national longitudinal open-cohort study was conducted using data from the Italian Veterinary Information System, including approximately 300,000 herd–year observations from 2022 to 2025. The outcome was the occurrence of at least one TB breakdown per herd–year, analyzed using a discrete-time hazard modeling approach based on a binomial generalized linear mixed model with province-level random effects. The incidence of TB remained very low but increased over time, and significant spatial clustering was observed. Higher TB risk was associated with larger herd size, a previous history of TB, non-OTF herd status, proximity to recent breakdowns, number of animals purchased, transhumance practices, and a shorter time since acquisition of OTF status at provincial level. These findings highlight that, even in disease-free contexts, TB risk is heterogeneous and driven by identifiable factors, supporting the refinement of targeted, risk-based surveillance to maintain OTF status over time.

## 1. Introduction

Animal tuberculosis in cattle (TB) is a chronic infectious disease caused by members of the *Mycobacterium tuberculosis* complex (MTBC), most notably *Mycobacterium bovis* and *Mycobacterium caprae* [1,2]. The infection typically evolves slowly, with a prolonged incubation period and often limited or absent clinical signs, while granulomatous lesions may develop primarily in the respiratory tract and associated lymph nodes [2]. Beyond its animal health and productivity impacts, TB remains relevant for public health because of the zoonotic potential of *M. bovis*, even though human disease is now relatively rare in the European Union (EU) context [1,2].

In the EU, long-standing eradication programs based on testing and removal of infected animals have markedly reduced TB prevalence, and extensive territories have achieved officially tuberculosis-free (OTF) status under EU legislation [2]. Nevertheless, the disease can persist or re-emerge through complex mechanisms that include residual infection, reintroductions via animal movements, and local ecological drivers, making sustained freedom particularly challenging in low-incidence or low-prevalence areas [2,3,4,5,6,7]. In fact, TB risk is shaped by factors operating at multiple levels—animal, herd, and area—and by the interaction between biological and non-biological determinants [2,3,7]. Among the most consistently reported drivers are cattle movements [5,6] and contacts with infected neighboring farms [4]. Additional determinants include changes in farming structure (e.g., increasing farm size and evolving husbandry practices) and the potential contribution of other domestic species and local wildlife population dynamics, whose relative importance may vary across epidemiological situations and over the course of control programs [7,8]. This context dependency is particularly relevant in regions with very low prevalence, where breakdowns are sporadic, transmission chains are short, and the marginal contribution of specific risk pathways may differ from endemic settings [9].

In low-incidence or disease-free areas, the efficiency of blanket surveillance strategies can decrease, while costs and operational burden increase; consequently, risk-based approaches have been proposed to better target surveillance toward herds and areas more likely to harbor infection [10,11]. In Italy, TB epidemiology has historically shown marked heterogeneity among territories, and EU has listed multiple Italian regions and provinces among OTF areas (Commission Implementing Regulation EU 2021/620). Italy has implemented a risk-based surveillance program in disease-free territories under current national provisions, which includes annual controls of all herds classified at higher risk according to predefined criteria. This activity is implemented alongside permanent slaughterhouse surveillance, which has consistently proved to be a key component in areas with a low TB incidence [12,13,14].

In this scenario, robust identification and prioritization of the most relevant risk factors becomes essential to optimize risk-based surveillance. Risk factors derived from endemic contexts or from other countries may not fully translate to Italian OTF regions because of differences in cattle demographics, production systems, movement patterns, and ecological interfaces. Therefore, generating evidence tailored to the Italian disease-free setting is critical to support surveillance planning and to maintain OTF status over time.

Following the entry into force of the EU Animal Health Law in April 2021, the definition of a confirmed TB case was revised. In Italy, herds were previously classified as infected based on positive tuberculin skin test results alone; currently, confirmation also requires the detection of tuberculous lesions and/or positive direct tests for MTBC (PCR or culture). For this reason, the present study included only TB breakdowns notified between 2022 and 2025. The aim of this study is to identify and evaluate the main risk factors associated with TB occurrence in OTF regions of Italy. By clarifying which drivers are most relevant in the Italian context, this work seeks to support the refinement of risk-based surveillance and contribute to the long-term sustainability of TB eradication in Italy.

## 2. Materials and Methods

### 2.1. Study Design and Population

A national longitudinal open-cohort study was conducted to identify risk factors for TB breakdowns in herds located in Italian OTF provinces, as classified on 31 December 2025. Data on bovine herds (cattle and buffalo) and TB breakdowns were obtained from the Italian Veterinary Information System portal (https://www.vetinfo.it/ accessed on 27 February 2026), managed by the Italian Ministry of Health. The unit of analysis was the herd–year pair over the period 2022–2025. Herds were considered at risk in year t if, by 31 December of the previous year (*t* − 1), there were no TB breakdowns, maintained a bovine population greater than zero, and belonged to one of the following production types: dairy, beef-fattening, beef-breeding, or mixed.

### 2.2. Outcome and Predictors

The outcome variable was defined as the occurrence of at least one TB breakdown during the observation year and was treated as a binary variable (0; 1). To ensure an appropriate temporal sequence between exposure and outcome, all covariates were measured with a one-year lag (*t* − 1). The predictors included the following groups of variables:

• Structural and management factors: Production type, management system, herd size, and animal movements (entries, exits, and slaughter rates).
• Spatial context: Presence of TB breakdowns and number of bovine herds within a 2 km radius of each herd.
• Disease history: Current herd health status and a binary indicator of TB occurrence within the previous five years (*t* − 1 to *t* − 5).

### 2.3. Statistical Analysis

Data were analyzed using a discrete-time hazard modeling approach implemented through a binomial generalized linear mixed model (GLMM) with a complementary log–log (cloglog) link function. This approach is particularly appropriate for modeling event occurrence in discrete follow-up intervals and provides a discrete-time analog of a continuous-time proportional hazards model [15,16].

Random intercepts were initially included at both herd and provincial levels to account for repeated measurements and unexplained territorial heterogeneity. Since the estimated herd-level variance resulted close to zero, a more parsimonious model including only province-level random effects was adopted. Multicollinearity among covariates was assessed using variance inflation factors (VIFs) and no evidence of problematic collinearity was detected (Supplementary Materials).

Model estimates are presented as discrete hazard ratios (HRs) with corresponding 95% Wald confidence intervals. In survival analysis, the HR quantifies the relative risk over time; specifically, it represents, within a defined follow-up period, the ratio of the hazard of an event in herds with a given factor compared with those without it. The variables N_Purchased, N_Sold and N_Herds_2 km were modeled on a log(1 + x) scale to account for non-linearity; therefore, the corresponding hazard ratio represents the effect of approximately a 2.7-fold increase in the original variable values. All statistical analyses were performed in R using the lme4 and tidyverse packages [17,18]. Figures were generated using ggplot2. Collinearity diagnostics were assessed using the performance package [19].

## 3. Results

About 300,000 herd–year pairs over the period 2022–2025 were analyzed in the study (Table 1). The variables investigated and the relative frequencies are shown in Table 2.

**Table 1:** Annual incidence of Bovine Tuberculosis in officially tuberculosis-free regions. Italy, 2022–2025.

Year	Total Herds (N)	TB Breakdowns (Cases)	Incidence	HR (95% CI) *	*p*
2022	79,271	17	0.02%	1.00 (ref.)	
2023	76,030	28	0.04%	2.86 (1.36–6.05)	0.0058
2024	73,111	37	0.05%	4.20 (1.93–9.16)	<0.001
2025	70,745	44	0.06%	5.76 (2.66–12.49)	<0.001
Total	299,157	126	0.04%		

**Figure 1:**
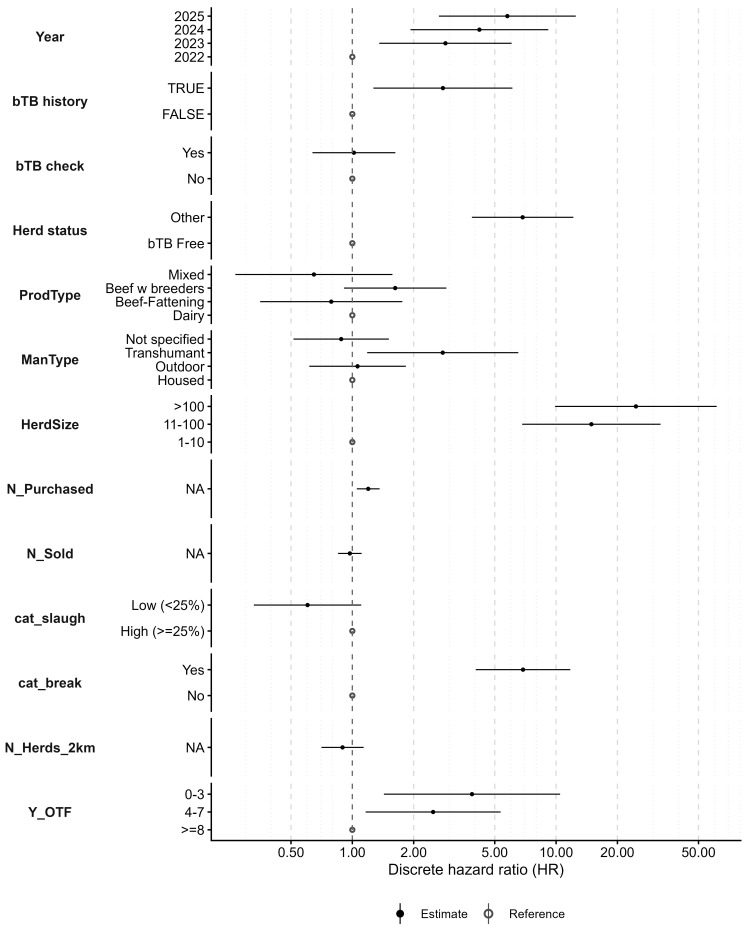
Hazard ratio estimates and corresponding 95% confidence intervals for the variables investigated. OTF provinces in Italy, 2022–2025. Full description of variables is reported in Table 2.

* HR (95% CI): hazard ratio (95% confidence interval). Hazard ratios are depicted in Figure 1.

**Table 2:** Variables investigated in officially tuberculosis-free regions. Italy, 2022–2025.

Variable	Variable Description	Range	No. of Events	Cases	Incidence	HR (95% CI) *	*p*
bTB history	A bTB breakdown occurred in the same herd in the previous 5 years	FALSE	298,852	115	0.04%	1.00 (ref.)	
		TRUE	305	11	3.61%	2.78 (1.27–6.11)	0.0106
bTB check	The herd had a bTB check in the previous year	NO	218,124	56	0.03%	1.00 (ref.)	
		YES	81,033	70	0.09%	1.02 (0.64–1.63)	0.9403
Herd status	Herd status in the previous year	bTB Free	254,147	101	0.04%	1.00 (ref.)	
		Other	45,010	25	0.06%	6.85 (3.86–12.15)	<0.001
ProdType	Herd production type	Dairy	82,288	23	0.03%	1.00 (ref.)	
		Beef-Fattening	93,562	26	0.03%	0.79 (0.35–1.76)	0.5612
		Beef-Breeding	84,944	69	0.08%	1.62 (0.91–2.89)	0.1
		Mixed	38,363	8	0.02%	0.65 (0.27–1.57)	0.3378
ManType	Management of the herd	Housed	92,294	36	0.04%	1.00 (ref.)	
		Outdoor	45,714	36	0.08%	1.06 (0.61–1.83)	0.8320
		Transhumant	10,322	9	0.09%	2.78 (1.18–6.53)	0.019
		Not specified	150,827	45	0.03%	0.88 (0.51–1.51)	0.6471
HerdSize	Herd size on 31 December of the previous year	1–10	132,417	9	0.01%	1.00 (ref.)	
		11–100	120,163	85	0.07%	14.90 (6.82–32.57)	<0.001
		>100	46,577	32	0.07%	24.64 (9.89–61.39)	<0.001
N_Purchased	Number of bovine heads purchased in the previous year	0–67,410	299,157	126	0.04%	1.20 (1.05–1.36)	0.006
N_Sold	Number of bovine heads sold in the previous year	0–65,988	299,157	126	0.04%	0.97 (0.85–1.11)	0.6807
cat_slaugh	Percentage of bovine heads slaughtered in the previous year	High (≥25%)	30,776	21	0.07%	1.00 (ref.)	
		Low (<25%)	268,381	105	0.04%	0.60 (0.33–1.11)	0.1021
cat_break	Proximity (2 km) with a bTB breakdown occurred in the previous 5 years	NO	295,810	95	0.03%	1.00 (ref.)	
		YES	3347	31	0.93%	6.88 (4.03–11.74)	<0.001
N_Herds_2 km	No. of bovine herds within 2 km	0–164	299,157	126	0.04%	0.90 (0.71–1.14)	0.3629
Y_OTF	Number of years since the province of the herd has held the OTF status	0–3	64,169	44	0.07%	3.87 (1.43–10.46)	0.0078
		4–7	81,317	29	0.04%	2.49 (1.16–5.34)	0.0190
		>7	153,671	53	0.03%	1.00 (ref.)	

* HR (95% CI): hazard ratio (95% confidence interval). Hazard ratios are depicted in Figure 1.

**Figure 2:**
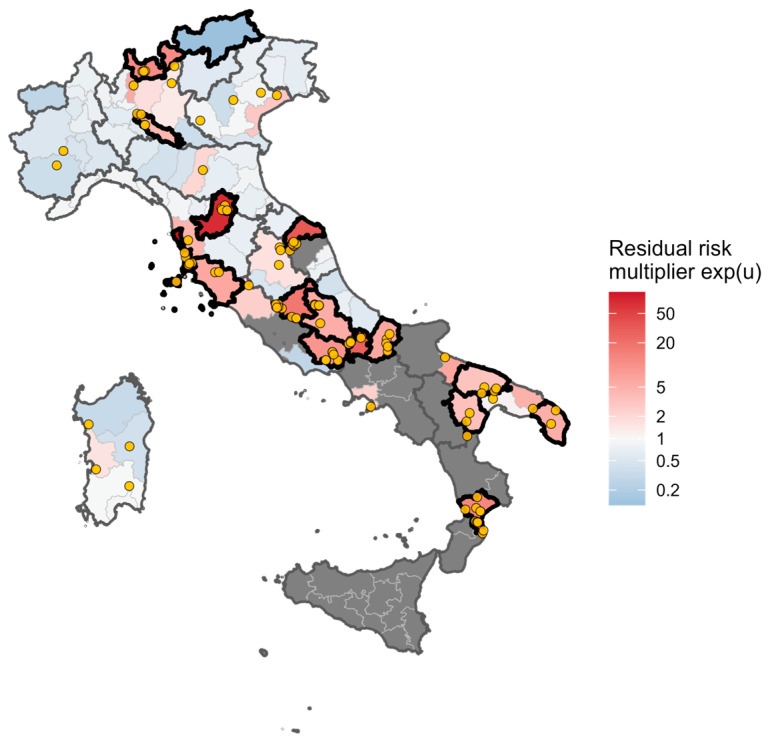
Provincial residual heterogeneity in TB breakdown risk in OTF provinces (2022–2025). Colors represent the province-level random intercept from the discrete-time hazard model (binomial GLMM with a complementary log–log link function), expressed as a residual risk multiplier exp(u) relative to the average province (exp(u) = 1). Provinces outlined in black have conditional 95% confidence intervals for exp(u) that do not include 1. Yellow points indicate the 126 herd–year TB breakdowns that occurred during the study period. Non-OTF provinces are gray.

During the 2022–2025 period, the incidence of TB progressively increased in disease-free provinces, reaching 0.06% by the end of 2025 (Table 1). Breakdowns appeared to be clustered at the provincial level (Figure 2), and the number of years since the acquisition of OTF status was also associated with the incidence of disease breakdowns. Compared with provinces that had maintained OTF status for more than seven years, provinces that had obtained OTF status less than three years earlier showed higher incidence rates (hazard ratio [HR] 3.96; 95% CI: 1.47–10.71), as did those that had been OTF for four to seven years (HR 2.43; 95% CI: 1.13–5.19).

Model results for the other variables investigated are reported in Table 2 and Figure 1. The distribution of nearby herds (within 2 km) was skewed, with median values of 10 (IQR: 5–19) for non-breakdown herd years and 6.5 (IQR: 3–11) for breakdown herd years.

Factors positively associated with TB incidence included herd size, the presence of a breakdown within a 2 km radius (HR 7.09; 95% CI: 4.14–12.15), a history of disease in the previous five years (HR 3.31; 95% CI: 1.52–7.25), and the absence of officially tuberculosis-free herd status (HR 6.95; 95% CI: 3.87–12.46).

Among herd management-related factors, the number of bovine animals purchased in the previous year (HR 1.20; 95% CI: 1.05–1.36) and the practice of transhumance (HR 2.51; 95% CI: 1.07–5.91) were identified as risk factors.

## 4. Discussion

To our knowledge, only a limited number of studies have investigated the factors associated with the occurrence of bovine TB in Italy, and even fewer have national relevance [20,21,22,23]. This investigation made it possible to identify those factors that are significant in Italian OTF zones.

Herd size has been frequently reported as a herd-level factor associated with the occurrence of TB, although its effect may vary according to the epidemiological context [5,24,25], also in low-incidence zones [23,26]. In this study, medium-sized (11–100 animals) and large herds (>100 animals) were at higher risk compared with small holdings, suggesting that the increase in risk is not limited to very large production systems but may emerge once a certain level of herd complexity is reached. The estimated hazard ratios were associated with wide confidence intervals, likely reflecting the small number of TB breakdowns, particularly within the reference group. This suggests limited precision in estimating the magnitude of the association, although its direction appears consistent. Larger herds are likely to experience higher internal contact rates and a greater probability of pathogen introduction through animal movements, which are recognized drivers of between-herd transmission [11,26]. Herd size is generally interpreted as a composite risk indicator, reflecting both biological processes related to transmission and operational aspects of testing and detection, rather than as an isolated causal factor.

Proximity to a confirmed TB breakdown was identified as a significant herd-level risk factor. This finding is consistent with several investigations showing that herds located close to infected holdings experience a higher probability of infection, reflecting short-range spread through direct or indirect contacts, shared pasture use, environmental contamination, or wildlife-mediated transmission [3,4,23,27]. Spatial clustering of breakdowns has been consistently reported in both endemic and officially tuberculosis-free regions [4,21,23], where re-emergence of infection is frequently driven by local rather than long-distance transmission processes. From a disease control perspective, these findings further support the relevance of spatially targeted approaches to TB surveillance and control. Testing of neighboring herds should be considered not only within the framework of annual risk-based surveillance planning, but also as a component of breakdown investigations following the detection of a new case of infection. Herds located in close proximity to recent breakdowns may warrant enhanced monitoring or the implementation of additional biosecurity measures, even in the absence of clear epidemiological links.

The presence of a random effect at the provincial level also seems to support the existence of local risk factors. In each TB breakdown, an epidemiological investigation is conducted [22], which sometimes identifies other related infected herds located at short distances, but not necessarily contiguous. These infection clusters reveal the presence of indirect links between breakdowns that were not accounted for in our study.

Also, a previous history of disease in the herd was associated with an increased risk of TB. This finding is consistent with several studies: Adkin et al. [26] identify herd history of TB breakdowns (years since the last episode) as the strongest predictor of future risk, while Bessell et al. [5] show that a previous history of TB breakdowns significantly increases the likelihood of subsequent breakdowns, particularly in high-incidence settings. Residual persistence, reinfections, or structural vulnerabilities that have not been fully resolved are often associated with the re-emergence of disease in herds that have been disease-free for only a few years [3,28], especially in Italy [22].

Transhumance, which involves seasonal movement to other regions—sometimes on foot—and grazing were associated with a higher risk of TB than other herd management types. This practice increases the likelihood of both direct and indirect contacts between herds and with the surrounding environment. This interpretation is consistent with several studies, which highlight the role of pasture use, local contacts and environmental exposure as important drivers of TB transmission, particularly in extensive or semi-extensive production systems [3,4,27,29]. Case–control studies conducted in officially tuberculosis-free (OTF) contexts in France emphasize local grazing contacts, shared pastures and other management-related factors as key determinants of infection, supporting the hypothesis that exposure at pasture may outweigh other risk factors in these settings [4].

In our study, no association was found between production type and an increased risk of TB, suggesting that the sporadic occurrence of TB in OTF areas is attributable to other factors. In fact, studies based on risk-based surveillance frameworks or modeling approaches in low-prevalence or OTF regions tend to highlight different drivers, such as animal movements, herd size and characteristics of the surveillance system, rather than production type per se. These studies primarily identify movements and herd size as the main determinants of TB risk, reflecting the dominant role of introduction pathways in low-incidence contexts [11,20,24,25]. Within this framework, production type may act as a proxy for underlying management characteristics—such as grazing, contact structure or biosecurity—rather than as an independent risk factor.

Bessell et al. [24] recommend annual testing of herds with a slaughter rate of <25% within risk-based surveillance frameworks, as these herds have a lower probability of infection detection through slaughterhouse surveillance. However, in the present study, a low slaughter rate (<25%) was not associated with a higher risk of TB.

In our study, incoming animal movements were associated with the occurrence of a TB breakdown. However, it should be noted that our analysis only considered whether animals had been introduced in the previous year. The introduction of infected animals may have occurred also in earlier years, which was not captured by our model. Animal movements, particularly in disease-free areas, are undoubtedly an important risk factor to consider; however, in Italian OTF areas the introduction of breeding animals is permitted only if they originate from OTF areas or countries, which substantially reduces the risk of disease introduction. This restriction does not apply to fattening herds, in which sporadic cases of TB are occasionally detected.

The TB eradication strategy in the EU is based on a test-and-cull approach and on the progressive assignment of disease-free status, first to herds and subsequently to territories. While the criteria for granting disease-free status to herds have essentially remained unchanged (two consecutive negative cervical tuberculin tests performed six months apart on all animals aged more than 42 days), since 2021, the criteria for assigning officially tuberculosis-free (OTF) status to an area have become less stringent. Previously, at least 99.9% of herds were required to be disease-free for seven consecutive years; currently, it is sufficient to have 99.8% of herds disease-free and an annual herd-level incidence below 0.1% for three consecutive years. Finally our study showed that the occurrence of TB was higher in herds that did not have TB-free status in the previous year, and in provinces that had acquired OTF status for less than seven years. This result may be related to residual infection persisting in the area, likely associated with the limited sensitivity of diagnostic tests, particularly in low-incidence settings [30].

## 5. Conclusions

Our findings support the inclusion of herd size, spatial proximity to a TB breakdown, a history of disease, and grazing among the variables used for risk-based surveillance and control strategies in the OTF regions of Italy. However, this type of surveillance is not recommended in zones that have acquired disease-free status for less than three years, in which it is advisable to continue annual testing of at least all herds with breeding animals.

## Data Availability

The authors do not have permission from the institution that funded the project to share farm-level data due to privacy concerns.

## Supplementary Materials

The following supporting information can be downloaded at https://www.mdpi.com/article/10.3390/pathogens15060636/s1; Text S1: Simplified R syntax of the final mixed-effect model; Table S1: Full fixed-effect estimates; Table S2: Variance components of the random effects; Table S3: Model fit statistics; Table S4: Variance inflation factors (VIFs) for fixed-effect predictors; Figure S1: Correlation matrix of the fixed-effect estimates.

## Abbreviations

The following abbreviations are used in this manuscript:

TB	Animal tuberculosis in cattle
EU	European Union
HR	Hazard Ratio
MTBC	*Mycobacterium tuberculosis* complex
OTF	Officially tuberculosis-free
