# Supplementary material for: A Study on Risk Factors for Bovine Tuberculosis in the Disease-Free Regions of Italy
Source: Pathogens. 2026 Jun 15;15(6):636. doi: 10.3390/pathogens15060636 (PMC13304961; doi:10.3390/pathogens15060636)
Supplement: Supplementary file 1 [file pathogens-15-00636-s001.zip › pathogens-4366694-supplementary.pdf]

# A study on risk factors for bovine tuberculosis in the disease-free regions of Italy

## Supplementary materials

The supplementary material reports the simplified R syntax used to estimate the binomial Generalized Linear Mixed Model (GLMM) with a complementary log-log (cloglog) link function, the full fixed-effect estimates, the random-effect variance components, the model-fit statistics, and the multicollinearity diagnostics for the fixed-effect structure. Multicollinearity was assessed using variance inflation factors (VIF). No evidence of problematic multicollinearity was detected.

In addition, Supplementary Figure S1 presents the correlation matrix of the fixed-effect estimates.

**Text S1.** Simplified R syntax of the final mixed-effects model

```
glmer(outbreak ~ fixed effects + (1 | province),
      family = binomial(link = "cloglog"),
      control = glmerControl(
        optimizer = "bobyqa",
        optCtrl = list(maxfun = 2e5)))
```

**Table S1.** Full fixed-effect estimates.

| Variable | Variable description | Range | Estimate | SE | z | p-value |
|---|---|---|---|---|---|---|
| **Intercept** | | | -12.8161 | 0.8555 | -14.981 | < 0.001 |
| **Year** | Year of breakdown occurrence | 2022 | | | | |
| | | 2023 | 1.0521 | 0.3817 | 2.757 | 0.00584 |
| | | 2024 | 1.4358 | 0.3973 | 3.614 | < 0.001 |
| | | 2025 | 1.7517 | 0.3947 | 4.438 | < 0.001 |
| **bTB history** | A bTB breakdown occurred in the same herd in the previous 5 years | FALSE | | | | |
| | | TRUE | 1.0236 | 0.4008 | 2.554 | 0.01065 |
| **bTB check** | The herd had a bTB checks in the previous year | NO | | | | |
| | | YES | 0.0179 | 0.2391 | 0.075 | 0.94027 |
| **Herd status** | Herd status in the previous year | bTB Free | | | | |
| | | Other | 1.9243 | 0.2925 | 6.579 | < 0.001 |
| **ProdType** | Herd production type | Dairy | | | | |
| | | Beef-Fattening | -0.2382 | 0.4099 | -0.581 | 0.56123 |

| | | | | | | |
|---|---|---|---|---|---|---|
| | | Beef-Breeding | 0.4843 | 0.2951 | 1.641 | 0.10078 |
| | | Mixed | -0.4341 | 0.4528 | -0.959 | 0.33777 |
| **ManType** | Management of the herd | Housed | | | | |
| | | Outdoor | 0.0591 | 0.2784 | 0.212 | 0.83201 |
| | | Transhumant | 1.0218 | 0.4358 | 2.345 | 0.01905 |
| | | Not specified | -0.126 | 0.2752 | -0.458 | 0.64713 |
| **HerdSize** | Herd size on 31 December of the previous year | 1-10 | | | | |
| | | 11-100 | 2.7013 | 0.399 | 6.77 | < 0.001 |
| | | >100 | 3.2045 | 0.4657 | 6.882 | < 0.001 |
| **N_Purchased** | Number of purchased bovine heads in the previous year | 0-67410 | 0.1795 | 0.0657 | 2.731 | 0.0063 |
| **N_Sold** | Number of sold bovine heads in the previous year | 0-65988 | -0.0279 | 0.0679 | -0.411 | 0.68074 |
| **cat_slaugh** | Percentage of bovine heads slaughtered in the previous year | High (≥25%) | | | | |
| | | Low (<25%) | -0.5061 | 0.3096 | -1.635 | 0.10214 |
| **cat_break** | Proximity (2 km) with a bTB breakdown occurred in the previous 5 years | NO | | | | |
| | | YES | 1.9288 | 0.2726 | 7.076 | < 0.001 |
| **N_Herds_2km** | No. of bovine Herds within 2km | 0-164 | -0.1106 | 0.1216 | -0.91 | 0.36294 |
| **Y_OTF** | Number of years since the province of the herd has held the OTF status | 0-3 | 1.3523 | 0.508 | 2.662 | 0.00777 |
| | | 4-7 | 0.9131 | 0.3893 | 2.346 | 0.01899 |
| | | >7 | | | | |

**Table S2.** Variance components of the random effects.

| Grouping factor | Term | Variance | Std. Dev. | Exp(SD) |
|---|---|---|---|---|
| Province | Intercept | 3.34 | 1.83 | 6.23 |

**Table S3.** Model fit statistics.

| Statistics | Value |
|---|---|
| AIC | 1696.5 |
| BIC | 1940.5 |
| logLik | -825.3 |
| No. observations | 299157 |
| No. provinces | 86 |
| Optimizer | bobyqa |

**Table S4.** Variance inflation factors (VIF) for fixed-effect predictors. VIF values greater than 10 are generally considered indicative of severe multicollinearity; values above 5 may suggest moderate collinearity requiring attention.

| Variable | Variable description | VIF | VIF 95% CI | SE inflation factor | Tolerance | Tolerance 95% CI |
|---|---|---|---|---|---|---|
| **Year** | Year of breakdown occurrence | 2.351 | 2.338–2.364 | 1.533 | 0.425 | 0.423–0.428 |
| **bTB history** | A bTB breakdown occurred in the same herd in the previous 5 years | 1.445 | 1.438–1.452 | 1.202 | 0.692 | 0.689–0.695 |
| **bTB check** | The herd had a bTB checks in the previous year | 1.265 | 1.259–1.270 | 1.125 | 0.791 | 0.787–0.794 |
| **Herd status** | Herd status in the previous year | 1.456 | 1.449–1.463 | 1.207 | 0.687 | 0.684–0.690 |
| **ProdType** | Herd production type | 2.374 | 2.361–2.388 | 1.541 | 0.421 | 0.419–0.424 |
| **ManType** | Management of the herd | 1.296 | 1.291–1.302 | 1.138 | 0.771 | 0.768–0.775 |
| **HerdSize** | Herd size on 31 December of the previous year | 1.322 | 1.316–1.328 | 1.15 | 0.756 | 0.753–0.760 |
| **cat_purc** | Purchasing of bovine heads in the previous year | 1.233 | 1.228–1.238 | 1.11 | 0.811 | 0.808–0.815 |
| **cat_sell** | Selling of bovine heads in the previous year | 1.401 | 1.395–1.408 | 1.184 | 0.714 | 0.710–0.717 |
| **cat_slaugh** | Percentage of bovine heads slaughtered in the previous year | 1.499 | 1.492–1.506 | 1.224 | 0.667 | 0.664–0.670 |
| **cat_break** | Proximity (2 km) with a bTB breakdown occurred in the previous 5 years | 1.513 | 1.505–1.520 | 1.23 | 0.661 | 0.658–0.664 |
| **N_Herds_2km** | No. of bovine Herds within 2km | 1.06 | 1.056–1.064 | 1.03 | 0.943 | 0.939–0.947 |
| **Y_OTF** | Number of years since the province of the herd has held the OTF status | 2.405 | 2.392–2.419 | 1.551 | 0.416 | 0.413–0.418 |

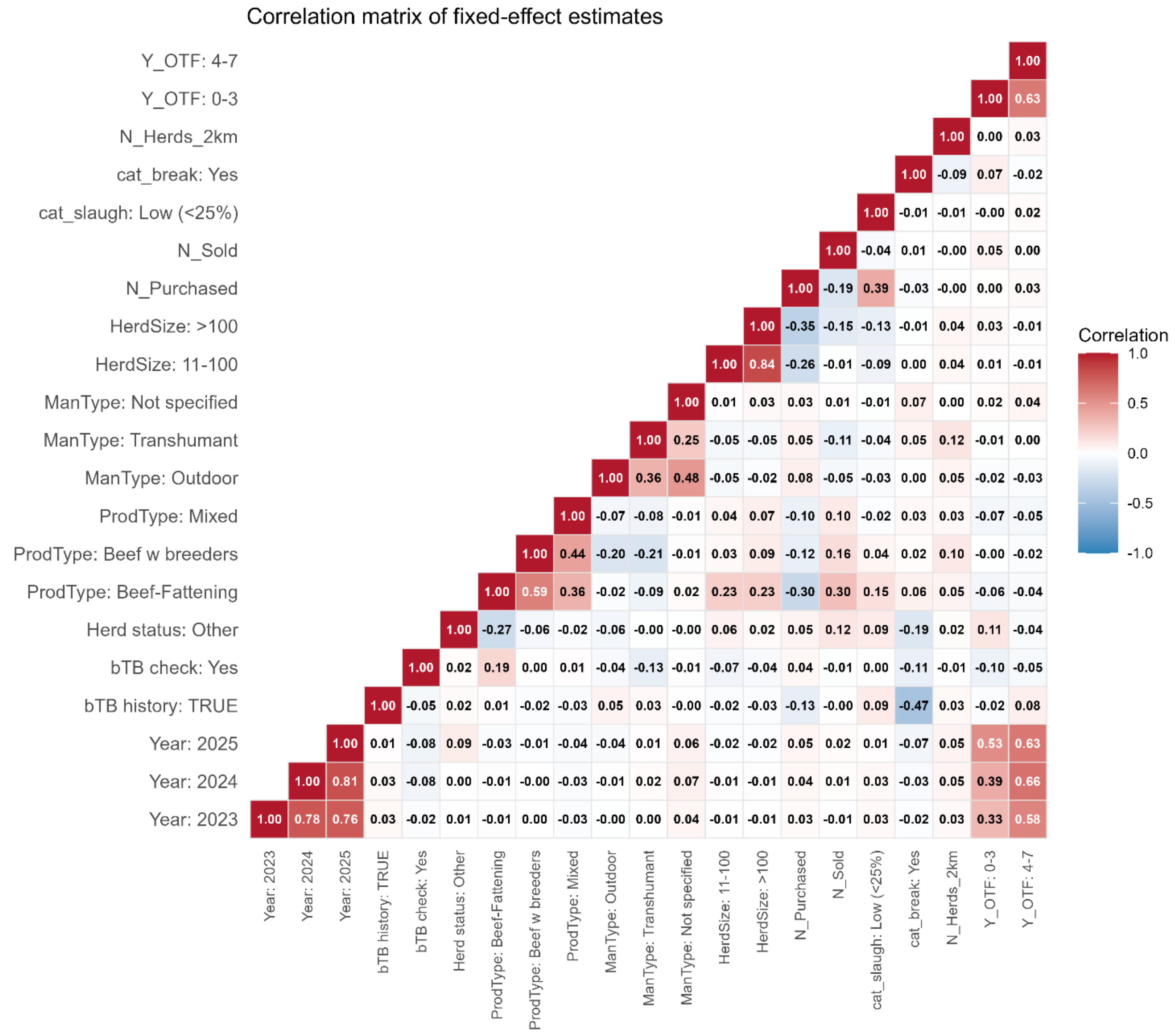


**Figure S1:** correlation matrix of the fixed-effect estimates
